# 25-Hydroxyvitamin D_3_ and 1,25-Dihydroxyvitamin D_3_ Promote the Differentiation of Human Subcutaneous Preadipocytes

**DOI:** 10.1371/journal.pone.0052171

**Published:** 2012-12-18

**Authors:** Hataikarn Nimitphong, Michael F. Holick, Susan K. Fried, Mi-Jeong Lee

**Affiliations:** 1 Department of Medicine, Section of Endocrinology and Metabolism, Ramathibodi Hospital, Bangkok, Thailand; 2 Department of Medicine, Section of Endocrinology, Diabetes and Nutrition, Boston University School of Medicine, Boston, Massachusetts, United States of America; Nihon University School of Medicine, Japan

## Abstract

1,25(OH)_2_D_3_ inhibits adipogenesis in mouse 3T3-L1 adipocytes, but little is known about its effects or local metabolism in human adipose tissue. We showed that vitamin D receptor (VDR) and 1α-hydroxylase (CYP27B1), the enzyme that activates 25(OH)D_3_ to 1,25(OH)_2_D_3_, were expressed in human adipose tissues, primary preadipocytes and newly-differentiated adipocytes. Preadipocytes and newly-differentiated adipocytes were responsive to 1,25(OH)_2_D_3_, as indicated by a markedly increased expression of CYP24A1, a primary VDR target. 1,25(OH)_2_D_3_ enhanced adipogenesis as determined by increased expression of adipogenic markers and triglyceride accumulation (50% to 150%). The magnitude of the effect was greater in the absence of thiazolidinediones. 1,25(OH)_2_D_3_ was equally effective when added after the removal of differentiation cocktail on day 3, but it had no effect when added only during the induction period (day 0–3), suggesting that 1,25(OH)_2_D_3_ promoted maturation. 25(OH)D_3_ also stimulated CYP24A1 expression and adipogenesis, most likely through its conversion to 1,25(OH)_2_D_3_. Consistent with this possibility, incubation of preadipocytes with 25(OH)D_3_ led to 1,25(OH)_2_D_3_ accumulation in the media. 1,25(OH)_2_D_3_ also enhanced adipogenesis in primary mouse preadipocytes. We conclude that vitamin D status may regulate human adipose tissue growth and remodeling.

## Introduction

In addition to its roles in regulating systemic calcium homeostasis and skeletal health, 1,25-dihydroxyvitamin D [1,25(OH)_2_D, D represents D_2_ or D_3_] regulates differentiation, proliferation and apoptosis of many cells types [1], [2]. Several studies showed that 1,25(OH)_2_D_3_ inhibits adipogenesis in 3T3-L1 cells [3], [4]. Although studies indicate that 1,25(OH)_2_D_3_ increases fatty acid synthetase activity in newly-differentiated human adipocytes [5], no previous studies addressed whether this hormone affects differentiation process in human preadipocytes. As the relevance of cultured mouse cell lines to human physiology is not known, we embarked on studies of 1,25(OH)_2_D_3_ action on the differentiation of primary human preadipocytes.

The local production of 1,25(OH)_2_D from 25-hydroxyvitamin D [25(OH)D], catalyzed by 1α-hydroxylase (CYP27B1), modulates the cell and tissue specific regulation of this hormone’s action [6], [7]. Previous studies demonstrated that VDR is expressed in human Simpson–Golabi–Behmel syndrome (SGBS) preadipocytes and adipocytes [8] and that 1α-hydroxylase is expressed in 3T3-L1 fibroblasts and rodent adipose tissues [9], but no data are available on intact human adipose tissue and its cellular constituents.

The first objective of this study was to determine whether the VDR and 1α-hydroxylase genes are expressed in human adipose tissues, in which cell types (adipocytes vs. stromal cells), and to assess how they are influenced by differentiation. The second objective was to assess the effects of both 25(OH)D_3_ and 1,25(OH)_2_D_3_ on early and late markers of adipogenesis [10], [11], and triglyceride accumulation in primary cultures of human subcutaneous preadipocytes.

## Materials and Methods

### Subjects

Adipose tissues were obtained from a total of 13 subjects during abdominal surgeries for severe obesity, gynecological abnormalities or panniculectomy. All subjects were free of diabetes, endocrine, or inflammatory diseases by medical history. Surgeries took place at the University of Maryland, School of Medicine, Baltimore, MD and Boston University, Medical Center, Boston, MA. All subjects gave informed consent as approved by IRB of the University of Maryland, School of Medicine and the Boston University, Medical Center.

### Measurement of VDR and CYP27B1 mRNA Expression in Human Adipose Tissues and Cell Fractions

Aliquots of adipose tissues were either immediately frozen in the operating room or transferred to the lab in Medium 199. Omental and subcutaneous adipose tissues from 4 subjects (3 females and one male with a mean age of 37.5±6.8 years and BMI 42±4.5 kg/m^2^) were used to prepare isolated adipocytes and stromal vascular cells (SVC) by collagenase digestion [12]. Total RNA was extracted from paired samples of tissue, isolated adipocytes and SVC and used to measure VDR and CPY27B1 mRNAs levels.

### Human Preadipocyte Culture and Differentiation

Abdominal subcutaneous adipose tissue samples from 9 subjects (8 females and one male) with a mean age of 44.8±3.5 years and BMI 32.8±8.2 kg/m^2^ (25.6–50.9) were used to prepare preadipocyte cultures by collagenase digestion [13], [14]. Stromal vascular cells were resuspended in growth media (α-MEM supplemented with 10% FBS, 100 units/ml penicillin, and 100 µg/ml streptomycin) and plated for culture. After subculturing 4 to 5 passages, cells were plated in 6 or 12 well plates (5000 cells/cm^2^) depending on the experimental design. For differentiation, 2d post-confluent cells (day 0) were treated with the adipogenic induction cocktail [DMEM/F12 with 500 µM 3-isobutyl-1-methylxanthine (IBMX), 100 nM human insulin, 100 nM dexamethasone, 1 µM thiazolidinedione (TZD, Rosiglitazone or in a few experiments, Ciglitazone), 2 nM T_3_, 10 µg/ml transferrin, 33 µM d-biotin, and 17 µM pantothenate] for 3 or 7 days [15]. After induction, cells were maintained in maintenance media [DMEM/F12 with 10 nM insulin and 10 nM dexamethasone]. There were no discernible differences in the results between the two types of TZD, so all data were pooled.

### Vitamin D Treatment

1,25(OH)_2_D_3_ (10^−10^, 10^−8^, 10^−7 ^M), 25(OH)D_3_ (10^−9^, 10^−8^ M) or ethanol (vehicle) was added continuously, only during the induction phase, or only during the maintenance phase, as specified in the figure legends. Preadipocytes from different subjects were not pooled. Independent experiments using cultures derived from the same individual provided consistent results. All experiments were repeated on cultures derived from at least 3 different subjects. We did not notice any variations in the effects of vitamin D as a function of the BMI of the donor. In separate experiments, we also tested the effects of 1,25(OH)_2_D_3_ in the absence of a TZD in the differentiation cocktail.

### 3T3-L1 Cell Culture

3T3-L1 fibroblasts were cultured in 10% FBS supplemented DMEM. 2d post-confluent (day 0) cells were differentiated in DMEM with 10% FBS, 500 µM IBMX, 100 nM bovine insulin, and 1 µM dexamethasone. Medium was replenished with DMEM+10% FBS with 100 nM insulin on d2, and with DMEM+10% FBS on d4. 1,25(OH)_2_D_3_ (10^−11^, 10^−10^, 10^−8^ M), 25(OH)D_3_ (10^−9 ^M), or vehicle (ethanol) was added to the media during differentiation at times specified in the figure legends.

### Mouse Primary Preadipocyte Culture and Differentiation

Stromal vascular cells from the inguinal adipose tissue of C57BL/6J mice were prepared as described for human preadipocytes. Cells were grown and differentiated as described for 3T3-L1 cells, except that the differentiation cocktail with Rosiglitazone (1 µM) was added only during the initial 2d-induction period. 1,25(OH)_2_D_3_ or vehicle control (ethanol) was added continuously until harvest on day 7. Animal studies were conducted in conformity with PHS policy and approved by IACUC of Boston University Medical Campus.

### Production of 1,25(OH)_2_D_3_ from 25(OH)D_3_

The ability of preadipocytes and newly-differentiated adipocytes to produce 1,25(OH)_2_D_3_ from 25(OH)D_3_ was tested. Upon reaching confluence, preadipocytes were incubated with 25(OH)D_3_ (10^−8^ M) for 24 h in α-MEM without FBS. Newly-differentiated human adipocytes were incubated with 25(OH)D_3_ (10^−8^ M) for 24 h in DMEM/F12 with no other additions. The quantity of 1,25(OH)_2_D_3_ in the incubation media was assayed with an enzyme immunoassay (Immunodiagnostic Systems Inc.). Data were expressed as picograms of 1,25(OH)_2_D_3_ produced per million cells.

### RNA Extraction and Measurement of Gene Expression

Total RNA was extracted using Trizol (Invitrogen) and quantity and quality were assessed spectrophotometrically. 1 µg total RNA was reverse transcribed using High-Capacity cDNA Reverse Transcription Kits (Applied Biosystems) and qPCR was performed with the Light Cycler 480 (Roche) with Taqman probes (Applied Biosystems). Cyclophilin A (PPIA) was used as a reference gene.

### Western Blotting

Cells were washed with ice-cold PBS and scraped in cell lysis buffer (Cell Signaling) supplemented with 5% SDS and protease inhibitors (Pierce). 5–10 µg total protein was resolved in 10 or 15% Tris-HCl gels (Biorad), transferred to PVDF membranes, and blocked in 5% milk in Tris buffered saline with 0.2% tween-20. Membranes were probed for FABP4 (a gift from Dr. Judith Storch at Rutgers University), VDR (D-6, Santa Cruz), adiponectin (BD Biosciences), CYP27B1 (C-12 and H-90, Santa Cruz), and loading controls [(α-tubulin (Santa Cruz) and total ERK (Cell Signaling)]. Chemiluminescence images were captured using an Imager (LAS 4000, Fuji) and quantified using software (Multi Guage, Fuji).

### Triglyceride (TG) and DNA Quantification

Total TG and DNA quantity in cell lysates were measured using a triglyceride determination kit (Sigma) and Quant-iT™ PicoGreen dsDNA reagent (Invitrogen).

#### Statistical analysis

Data are expressed as means±standard error mean (SEM). After log transformation, the differences between groups were determined by analysis of variance with repeated measures and 2-tailed Student t tests using GraphPad (GraphPad Software). Means were considered statistically different when p values were less than 0.05.

## Results

### VDR and CYP27B1 Genes are Expressed in Omental and Subcutaneous Human Adipose Tissues and Primary Preadipocytes and Adipocytes

**Figure 1:**
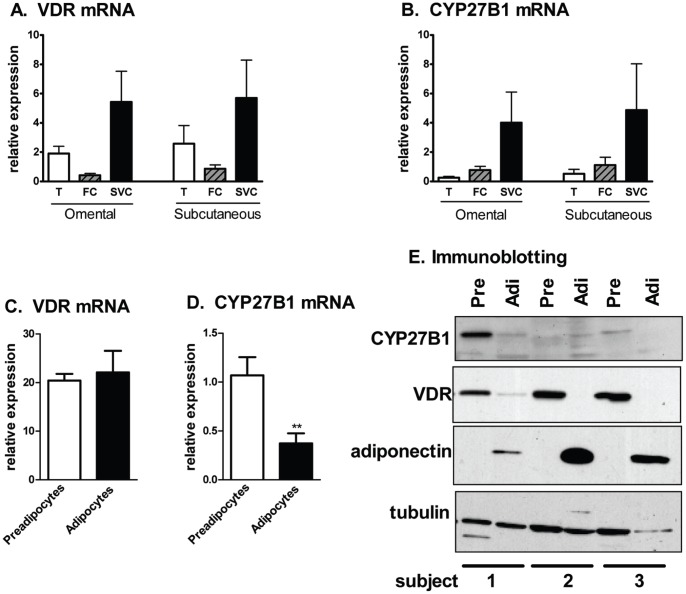
Expression levels of VDR and CYP27B1 in human adipose tissues and primary cultures of human preadipocytes and adipocytes. A and B. Expression levels of VDR and CYP27B1 mRNA were measured in adipose tissue (T), isolated fat cells (FC) and stromal vascular cells (SVC) from human omental and subcutaneous depots (n = 4). C and D. Expression levels of VDR and CYP27B1 mRNA were measured in human preadipocytes and newly-differentiated adipocytes (n = 5). **, p<0.01, preadipocytes vs. adipocytes. E. Protein levels of CYP27B1, VDR and adiponectin were measured with immunoblotting in 3 independent subjects before (preadipocytes; Pre) and 14d after differentiation (adipocytes: Adi).

VDR and CYP27B1 (1α-hydroxylase) mRNA were easily detected in samples of both omental and sc human adipose tissues (Fig. 1A,B). Expression levels of these mRNAs were similar between the two depots and were enriched in the stromal vascular cell compared to mature adipocyte fraction.

We next determined whether VDR and CYP27B1 expression levels varied with preadipocyte differentiation. VDR mRNA levels did not change after differentiation, while VDR protein levels decreased (Fig. 1C,E). CYP27B1 mRNA levels decreased after differentiation, but due to low expression in some samples, we were unable to demonstrate consistent changes in CYP27B1 protein levels (Fig. 1D,E).

**Figure 2:**
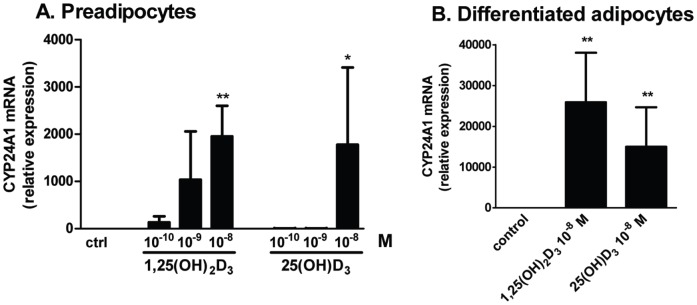
1,25(OH)_2_D_3_ and 25(OH)D_3_ increased CYP24A1 mRNA in preadipocytes and newly-differentiated adipocytes. A. Preadipocytes were treated with vehicle control, 1,25(OH)_2_D_3_ (10^−10^, 10^−9^, 10^−8^ M), or 25(OH)D_3_ (10^−10^, 10^−9^, 10^−8^ M) for 24 h and CYP24A1 mRNA expression was measured (n = 3). B. Differentiated adipocytes were treated with vehicle control, 1,25(OH)_2_D_3_ (10^−8^ M), or 25(OH)D_3_ (10^−8^ M) for 24 h and CYP24A1 mRNA expression was measured (n = 5). **, p<0.01, control vs. treatments.

To determine whether human preadipocytes and adipocytes respond to 1,25(OH)_2_D_3_, we tested whether it increased the expression of a known vitamin D target gene, CYP24A1. 1,25(OH)_2_D_3_ markedly increased CYP24A1 mRNA in both human preadipocytes and newly-differentiated adipocytes (Fig. 2). In addition, 25(OH)D_3_ induced CYP24A1 mRNA expression in both human preadipocytes and newly-differentiated adipocytes.

### Both 1,25(OH)_2_D_3_ and 25(OH)D_3_ Increased the Differentiation of Human Preadipocytes

**Figure 3:**
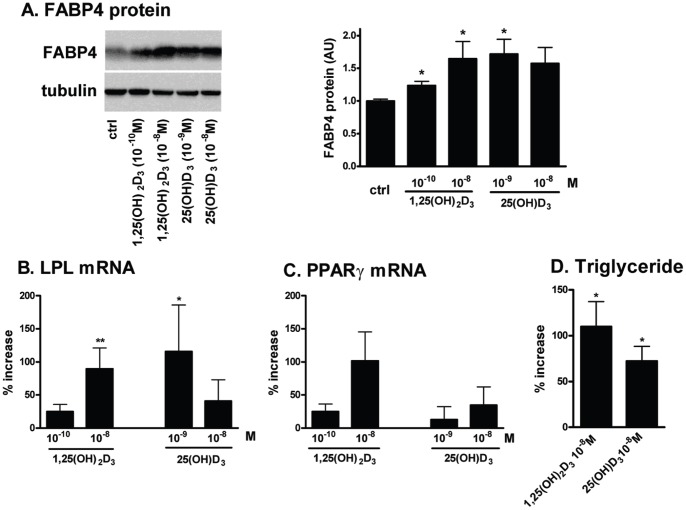
25(OH)D_3_ and 1,25(OH)_2_D_3_ promoted the differentiation of human preadipocytes. Human preadipocytes were differentiated in the presence of vehicle control, 1,25(OH)_2_D_3_ (10^−10^, 10^−8 ^M) or 25(OH)D_3_ (10^−9^, 10^−8 ^M) and expression levels of adipogenic markers were measured on d14. A. Representative immunoblots of FABP4 protein (left panel) and quantification (right panel) are shown (n = 6). Expression levels of LPL (B; n = 7) and PPARγ mRNA (C; n = 6) and TG accumulation (D; n = 4) were presented as % increase over vehicle control. *, p<0.05, **, p<0.01, vehicle control vs. treatments.

To test the effects of 1,25(OH)_2_D_3_ on human preadipocyte differentiation, 2d-post confluent preadipocytes were differentiated in the absence or presence of 1,25(OH)_2_D_3_ (10^−10^, 10^−9^, 10^−8^ M, added continuously throughout). 1,25(OH)_2_D_3_ dose-dependently enhanced adipogenesis as determined by significant increases in the expression levels of adipogenic markers (FABP4 protein and LPL mRNA) and TG accumulation (Fig. 3). 1,25(OH)_2_D_3_ (10^−8^ M) also tended to increase PPARγ mRNA levels in this dose-response experiment (p = 0.06, n = 6). A statistically significant effect of 1,25(OH)_2_D_3_ (10^−8^ M) to increase PPARγ mRNA levels was clear when these data and those from other experiments, also conducted at 10^−8^ M with the identical protocol, were combined (n = 9, p = 0.02). 1,25(OH)_2_D_3_ treatment did not affect the number of cells per well (not shown).

Since 25(OH)D_3_ also increased CYP24A1 expression, the effects of 25(OH)D_3_ on adipogenesis were tested. 25(OH)D_3_ increased differentiation of human preadipocytes (Fig. 3). Interestingly, 10^−8^ M 25(OH)D_3_ tended to be less effective than 10^−9^ M at increasing LPL mRNA and FABP4 protein levels. Of note, we could not test higher concentrations of 25(OH)D_3_ (≥10^−7^ M) as they were toxic to human preadipocytes, killing cells within 24 h of treatment. 25(OH)D_3_ (10^−8^ M) significantly increased triglyceride accumulation by 72±16% compared to the vehicle control (Fig. 3D).

### 1,25(OH)_2_D_3_ Primarily Regulates the Late Stage of Adipogenesis

**Figure 4:**
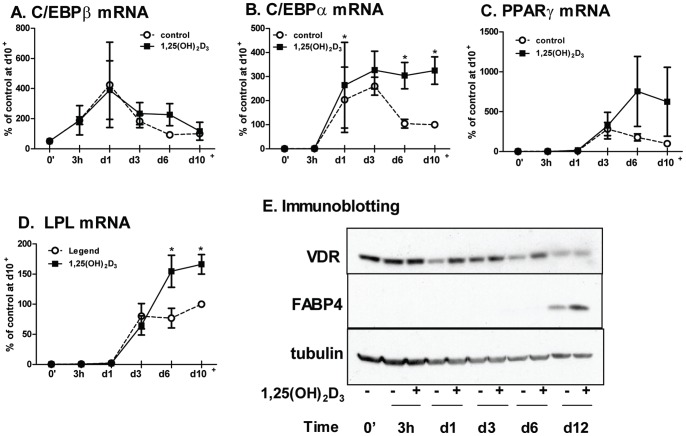
Time-course effects of 1,25(OH)_2_D_3_ on adipogenic marker expression. Human preadipocytes were differentiated in the absence or presence of 1,25(OH)_2_D_3_ (10^−8^ M, added continuously throughout). Expression levels of adipogenic markers [C/EBPβ (A), C/EBPα (B), PPARγ (C), and LPL (D)] were measured before (0′) and at indicated time points during differentiation. Data are presented as % of vehicle control after differentiation (d10–12; d10+) in each experiment. *, p<0.05, vehicle control vs. 1,25(OH)_2_D_3_ treatment, n = 4. E. Representative FABP4 and VDR blots from 3 independent experiments are presented.

To determine whether 1,25(OH)_2_D_3_ affects early or late events in adipogenesis, we next assessed the time course effects of 1,25(OH)_2_D_3_ on mRNA levels of key transcription factors and adipocyte genes during differentiation [10], [11]. 1,25(OH)_2_D_3_ did not affect mRNA levels of C/EBPβ, an early adipogenic transcription factor [16], [17] (Fig. 4A). However, 1,25(OH)_2_D_3_ significantly increased C/EBPα by ∼60% above the vehicle control on day 1 (Fig. 4B). Intriguingly, while C/EBPα expression declined after day 3 in controls, higher expression was maintained throughout differentiation in the 1,25(OH)_2_D_3_-treated cells. Thus, between day 6–10 of differentiation C/EBPα expression levels were 2 to 3-fold higher in the 1,25(OH)_2_D_3_-treated cells. Similar results were observed for PPARγ mRNA, although the difference was not statistically significant (Fig. 4C). 1,25(OH)_2_D_3_ increased LPL mRNA (a late marker of adipogenesis) only during the later period of differentiation (day 6+) (Fig. 4D). Similar data was obtained for FABP4 protein (Fig. 4E) and adiponectin mRNA levels (not shown), other late markers of adipogenesis. Although VDR mRNA levels remained unchanged throughout differentiation (not shown), VDR protein levels are decreased after differentiation (Fig. 4E). The rate of decline in VDR protein during differentiation was consistently slower when 1,25(OH)_2_D_3_ was added.

**Figure 5:**
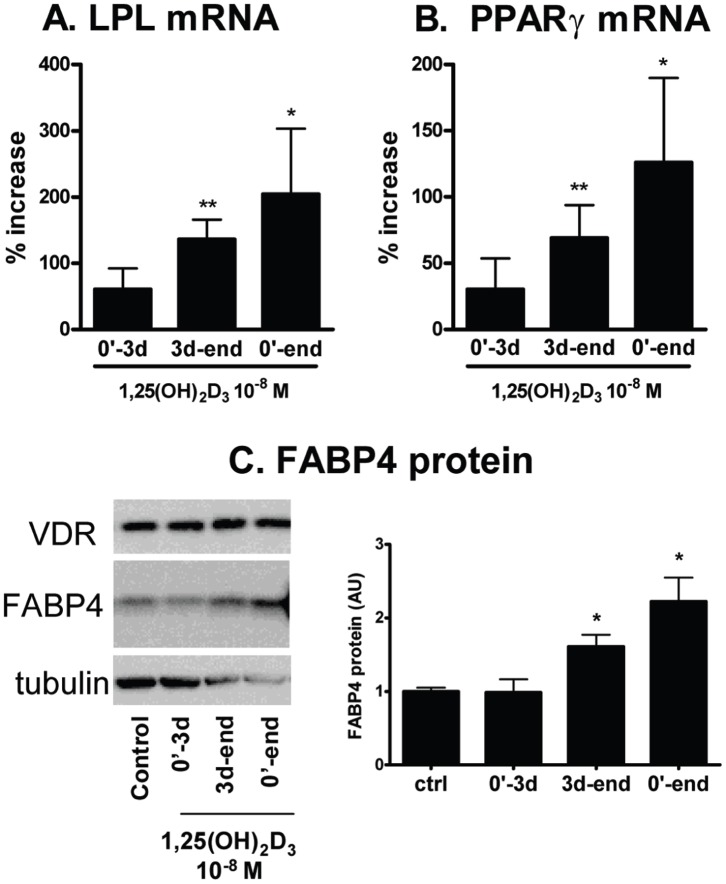
1,25(OH)_2_D_3_ promoted the maturation phase of adipogenesis. Human preadipocytes were differentiated in the adipogenic cocktail for 3 days and then maintained in the maintenance media until harvest (d13–14). 1,25(OH)_2_D_3_ (10^−8^ M) was added during the first 3 days of induction (0′–3d), maturation (3d-end), or continuously throughout (0′-end). Expression levels of adipogenic markers [LPL (A, n = 6) and PPARγ (B, n = 6) mRNA and FABP4 protein (C, n = 4)] were measured after differentiation. Data are presented as % increase over vehicle control. *, p<0.05, **, p<0.01, vehicle control vs. 1,25(OH)_2_D_3_ treatment.

To test whether 1,25(OH)_2_D_3_ affected the induction or maturation phase of adipogenesis, 1,25(OH)_2_D_3_ (10^−8^ M) was added continuously from the start of differentiation (0′-end), only during the initial 3d-induction period (0′–d3), or between day 3 to day 14 (d3-end). When added during the induction period (0′–3d), 1,25(OH)_2_D_3_ did not significantly affect the expression of any differentiation markers (Fig. 5). On the other hand, addition of 1,25(OH)_2_D_3_ during the maturation period (d3-end) significantly increased differentiation to the same extent as the continuous treatment (0′-end).

### The Pro-adipogenic Effects of 1,25(OH)_2_D_3_ are Greater in the Absence of Thiazolidinediones (TZD)

**Figure 6:**
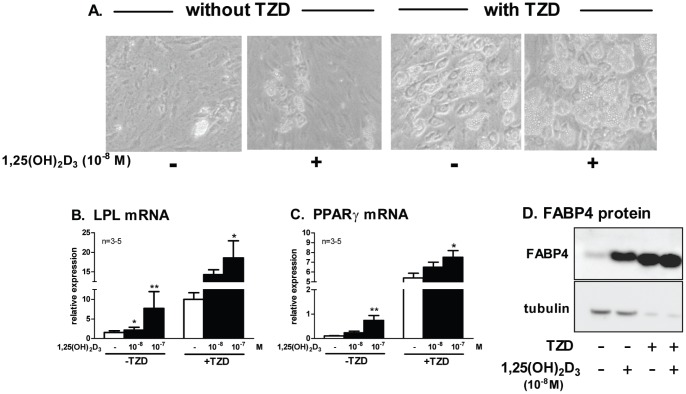
The pro-adipogenic effects of 1,25(OH)_2_D_3_ were independent of thiazolidinedione treatment. Human preadipocytes were differentiated in the differentiation cocktail with or without thiazolidinedione (TZD) for 7 days and maintained in maintenance media until harvest. 1,25(OH)_2_D_3_ or vehicle control was present throughout. Phase contrast image of adipocytes were taken at day 13 after differentiation (A). Expression levels of adipogenic markers [LPL (B) and PPARγ (C) mRNA and FABP4 (D) protein] were measured after differentiation (d13–14). Lane 3 and 4 (differentiated in the presence of TZD) were intentionally under loaded to show the results in the same blot. *, p<0.05, **, p<0.01, vehicle control vs. 1,25(OH)_2_D_3_ treatment, n = 3 for 10^−8^ and n = 5 for 10^−7 ^M.

Previous studies indicate that TZD partially ameliorate the inhibitory effects of vitamin D on adipogenesis [4], [18]. Since a TZD was one of regular components in our differentiation cocktail and TZDs are potent stimulators of adipogenesis [19], we also tested the effects of 1,25(OH)_2_D_3_ in the absence of a TZD. As expected, without TZD fewer cells accumulated lipid (Fig. 6A). Notably however, the magnitude of induction of adipogenic markers by 1,25(OH)_2_D_3_ (fold stimulation) was greater in the absence of a TZD (Fig. 6B–D).

### Activation of 25(OH)D_3_ in Human Preadipocytes

Because CYP27B1 expression was detectable and 25(OH)D_3_ induced CYP24A1 expression, we conducted preliminary studies to determine whether the enzyme was active. Preadipocytes incubated with 25(OH)D_3_ (10^−8 ^M, 24 h) produced detectable quantities of 1,25(OH)_2_D_3_ in the media. 4 samples tested produced 48±20 pg/10^6^ cells and one sample made much higher amounts, 1600 pg/10^6^ cells. In newly-differentiated adipocytes, only 2 out of 5 samples tested produced detectable amounts of 1,25(OH)_2_D_3_ (47 and 67 pg/10^6^ cells).

### In 3T3-L1 Preadipocytes, 1,25(OH)_2_D_3_ Inhibited Adipogenesis while 25(OH)D_3_ had No Effect

**Figure 7:**
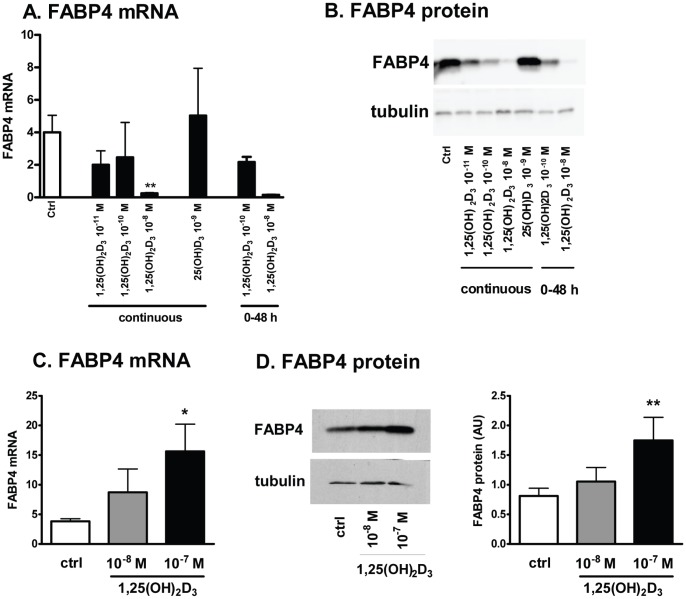
Effects of 1,25(OH)_2_D_3_ on differentiation of 3T3-L1 preadipocytes (A & B) and mouse preadipocytes (C & D). A&B. 3T3-L1 cells were grown and differentiated using a standard protocol. Vehicle control, 1,25(OH)_2_D_3_ or 25(OH)D_3_ was added at indicated doses or periods of differentiation. FABP4 expression levels were measured as a late marker of differentiation. **, p<0.01, control vs. treatment, n = 2–3. C& D. 2d-post confluent mouse preadipocytes were differentiated in the presence of thiazolidinedione (1 µM Rosiglitazone during 2d-induction period). 1,25(OH)_2_D_3_ (10^−8^, 10^−7 ^M) was added continuously and the degree of differentiation was determined by measuring FABP4 expression levels after differentiation. *, p<0.05, vehicle control vs. treatment, n = 4.

We tested the effects of 1,25(OH)_2_D_3_ on 3T3-L1 adipogenesis to determine if we could confirm its reported inhibitory effects [3], [4], [20]. Previous studies had detected 1α-hydroxylase activity in 3T3-L1 preadipocytes [9], yet none had tested the effects of 25(OH)D_3_ on adipogenesis in 3T3-L1 cells. In 3T3-L1 cells, 1,25(OH)_2_D_3_ caused a dose- and time-dependent inhibition of adipogenesis (Fig. 7A&B), as previously documented [3], [4]. Additionally, in contrast to its pro-adipogenic effects in human preadipocytes, 25(OH)D_3_ did not affect adipogenesis in 3T3-L1 cells (as shown by the lack of change in FABP4 expression levels, Fig. 7A&B).

To evaluate the possibility that apparent species differences between human preadipocytes and 3T3-L1 cells were not merely related to the initial level of commitment to the adipocyte cell fate, we also tested the effect of 1,25(OH)_2_D_3_ on primary mouse preadipocyte differentiation. 1,25(OH)_2_D_3_ increased the differentiation of mouse preadipocytes as determined by increases in FABP4 (Fig. 7C&D) and other markers of adipogenesis (adiponectin and PPARγ mRNA, not shown).

## Discussion

Our findings provide a number of novel insights into vitamin D actions on human adipose tissue. In contrast to its inhibitory effects in a mouse preadipocyte cell line, 3T3-L1, 1,25(OH)_2_D_3_ promoted adipogenesis in primary human preadipocytes as evidenced by the increased expression of adipogenic markers and lipid filling. In addition, we show that 25(OH)D_3_ can also promote the differentiation of human adipocytes, most likely via its activation to 1,25(OH)_2_D_3_. Furthermore, 1,25(OH)_2_D_3_ also had stimulatory effects on the differentiation of primary mouse preadipocytes. These results suggest that the local metabolism of vitamin D in adipose tissue may regulate the conversion of preadipocytes to adipocytes and hence support the healthy remodeling of human adipose tissue.

Addition of 1,25(OH)_2_D_3_ to the standard differentiation cocktail promoted the maturation of adipogenesis. Although 1,25(OH)_2_D_3_ did not affect the expression of C/EBPβ, an early marker of adipogenesis, it led to sustained increases in C/EBPα and PPARγ gene expression during the late phase of differentiation. Thus, 1,25(OH)_2_D_3_ may promote the differentiation of human preadipocytes by maintaining a high expression level of these key adipogenic transcription factors [10], [11]. It is notable that 1,25(OH)_2_D_3_ increased adipocyte maturation by 50–150% even when added in the presence of a TZD, which has a strong stimulatory effect on adipogenesis, suggesting that activation of these two signaling pathways has additive effects on adipogenesis. Not surprisingly, we found that the magnitude of the stimulatory effect of 1,25(OH)_2_D_3_ on adipogenesis was greater when it was added in the absence of TZD. These data suggest that the action of 1,25(OH)_2_D_3_ on adipogenesis can be independent of the activation of the PPARγ pathway, although the influence of Vitamin D on the production of an endogenous ligand for PPARγ cannot be ruled out. Further research that dissects the molecular mechanisms mediating Vitamin D actions on adipogenesis is needed.

Our data demonstrating that 1,25(OH)_2_D_3_ and 25(OH)D_3_ enhanced human preadipocyte differentiation are consistent with the findings that VDR−/− mice are leaner and resistant to diet induced obesity [21], [22]. CYP27B1 (1α-hydroxylase)−/− mice also have a lean phenotype [21]. Similarly, mice engineered to overexpress VDR in both white and brown adipose tissue are obese, and had similar food intake and lower energy expenditure per gram body weight [23]. Although the phenotypes of these transgenic mouse models have been attributed to alterations in energy expenditure, this conclusion is mainly based on the expression of metabolic rates divided by body weight, which is now considered inappropriate when % fat differs in two groups [24], [25]. Further, apparent alterations in white adipose metabolism in the adipose VDR overexpressors could be secondary to the obesity itself, and are difficult to evaluate without data on adipocyte size. In the VDR knockouts, the size rather than the number of adipocytes was affected, consistent with our data showing that 1,25(OH)_2_D_3_ may promote maturation/lipid filling rather than acting on the induction of adipogenesis or proliferation, which would affect number of adipocytes. Further studies of the in vivo consequences of altering VDR levels in white adipocytes only will be of great interest.

Similar to our results that show an increase in lipid accumulation, Li et al showed that 1,25(OH)_2_D_3_ increases lipoprotein lipase expression in 3T3-L1 preadipocytes [9]. In addition, Shi et al found that 1,25(OH)_2_D_3_ increases the enzymatic activities of fatty acid synthetase and GAPDH through non-genomic actions in newly-differentiated human adipocytes [5]. Studies that address genomic and non-genomic mechanisms by which vitamin D promote preadipocyte maturation and lipid filling are needed.

Although 25(OH)D_3_ increased adipogenesis and induced CYP24A1 mRNA to a similar extent as 1,25(OH)_2_D_3_, our study cannot definitively establish whether this is due to the conversion of 25(OH)D_3_ to 1,25(OH)_2_D_3_. It is generally assumed that the induction of CYP24A1 mRNA, is due to the genomic actions of VDR, presumably by 1,25(OH)_2_D [26]. However, non-genomic mechanisms can not be ruled out. Further, at high concentrations, 25(OH)D_3_ can also directly induce CYP24A1 gene expression in several cell types [26], [27], although the physiological relevance is unclear. Regardless of the mechanism involved, our observations indicate that low vitamin D status in obesity may have implications for adipose tissue biology that merit further study.

The results of this study demonstrate for the first time that CYP27B1 mRNA, which encodes the 1α-hydroxylase that converts 25(OH)D to the biologically active 1,25(OH)_2_D, was present at significant levels in both omental and subcutaneous human adipose tissues. This gene was mainly expressed in the stromal vascular fraction of human adipose tissue that contains preadipocytes, macrophages and endothelial cells. CYP27B1 is known to be expressed in macrophages and endothelial cells, so this result is not unexpected [28]–[30]. In addition, CYP27B1 was also expressed in cultures of human primary preadipocytes and newly-differentiated adipocytes, which lack of macrophage or endothelial markers [14]. Moreover, we found that 1,25(OH)_2_D_3_ synthesis was consistently detectable in preadipocyte cultures incubated with 25(OH)D_3_. Consistent with this result, Ching et al recently reported that mammary preadipocytes and adipocytes can synthesize 1,25(OH)_2_D_3_ from 25(OH)D_3_ [31]. Additionally, we noted subject dependent variability in the activation of 25(OH)D_3_ to 1,25(OH)_2_D_3_ in both preadipocytes and adipocytes (15 to 1600 pg/10^6^ cells).

In preliminary experiments we found that intact human adipose tissue fragments produced easily detectable quantities of 1,25(OH)_2_D_3_ from 25(OH)D_3_ (HN and MJL, unpublished observation). Because adipose tissues of obese are infiltrated with macrophages, it seems likely that macrophages also contribute to the local activation of vitamin D. Further studies are needed to pinpoint the relative contribution of different cell type(s) expressing 1α-hydroxylase in human adipose tissues and to determine how vitamin D activation may change with pathophysiological states such as obesity. Nevertheless, the current results are consistent with the idea that 25(OH)D is activated locally within human adipose tissue and provide strong motivation for further studies directed at understanding the physiological and pathophysiological importance of local 1,25(OH)_2_D_3_ production in amplifying vitamin D action in human adipose tissues.

In contrast to our results that 1,25(OH)_2_D_3_ promoted human preadipocyte differentiation, Lorente-Cebrian recently noted that they could not find any effect on differentiation [32]. Unfortunately, details such as the timing of addition of the hormone were not provided. Another recent study reported that 25(OH)D_3_ and 1,25(OH)_2_D_3_ transiently suppresses differentiation of human mammary preadipocytes, as assessed by Oil Red O staining, during an early stage (day 7), but had no effect at a later stage (day 14) [31]. This discrepancy could be due to possible depot differences in response to 1,25(OH)_2_D_3_ treatment.

The pro-adipogenic effect of 1,25(OH)_2_D_3_ in human preadipocytes is in contrast to its anti-adipogenic effect in the commonly used preadipocyte cell line, 3T3-L1 [3], [4], [20]. Kong et al showed that exposure of 3T3-L1 to 1,25(OH)_2_D_3_ during the initial 2d-induction period is critical for its inhibitory action [4] and we also confirmed this in the current study. In human preadipocytes, 1,25(OH)_2_D_3_ was not effective in increasing adipogenesis when added during the 3d-induction period, while addition of 1,25(OH)_2_D_3_ during the maturation phase produced the same stimulation of adipogenesis as the continuous treatment. The difference between human primary preadipocytes and mouse 3T3-L1 cells may be related to the fact that human preadipocytes are at a more advanced stage of differentiation. Consistent with this idea, we found that 1,25(OH)_2_D_3_ also increased adipogenesis in primary mouse preadipocytes, which are also considered to be at least partially committed to an adipocyte cell fate.

In conclusion, our studies provide evidence that 1,25(OH)_2_D_3_ as well as 25(OH)D_3_ can influence human adipocyte differentiation by acting during the maturation and lipid filling processes. Although the mechanisms by which 25(OH)D and 1,25(OH)_2_D influence human adipogenesis require further investigation, we speculate that vitamin D actions may promote the healthy remodeling of adipose tissue as dying adipocytes are replaced with newly-differentiated, insulin-sensitive ones [33], similar to the actions of TZDs [19]. Given evidence from clinical and epidemiological studies implicating low vitamin D status in inflammation and insulin resistance in obesity, and as a predictor of development of Type 2 Diabetes [34]–[37], the current results provide a strong rationale for further studies of the molecular mechanisms that regulate vitamin D metabolism and action in human adipose tissue, adipocytes and preadipocytes.
